# Epidemiological and Clinical Features of Severe Fever with Thrombocytopenia Syndrome during an Outbreak in South Korea, 2013–2015

**DOI:** 10.4269/ajtmh.16-0251

**Published:** 2016-12-07

**Authors:** Sun-Whan Park, Jungsang Ryou, Woo-Young Choi, Myung-Guk Han, Won-Ja Lee

**Affiliations:** 1 Korea Centers for Disease Control and Prevention, Cheongju, Republic of Korea

## Abstract

Since the first reported case of severe fever with thrombocytopenia syndrome (SFTS) in South Korea in 2013, between 2013 and 2015, we collected 1,697 serum samples from suspected patients who experienced symptoms of SFTS. We performed reverse transcriptase polymerase chain reaction using total RNA extracted from the patients' sera. When viral RNA was detected in the sera, SFTS was diagnosed. Among the 1,697 samples, 170 were positive for SFTS virus. We then analyzed the epidemiologic features of these 170 cases. As a result, we found that the annual number of cases increased steadily. However, the annual case fatality rate exhibited a downward trend. The majority of patients were aged ≥ 60 years, and most cases occurred during May–October in the eastern and southern parts of the country. These results may be useful for effective SFTS control by describing the clinical and epidemiologic features of the disease in South Korea.

Severe fever with thrombocytopenia syndrome (SFTS) is a new emerging infectious disease in China, which is caused by the SFTS virus (SFTSV), a phlebovirus in the family *Bunyaviridae*. The major clinical symptoms and laboratory findings of SFTS include fever, thrombocytopenia, gastrointestinal symptoms, leukopenia, and elevated serum hepatic enzyme levels. Patients with SFTS usually die due to multiple organ failure, with an average case fatality rate of 12% and up to 30% in some areas.^1^ SFTS was first reported in China^1^ and more recently in Japan^2^ and Korea.^3^,^4^ In addition, two cases with comparable symptoms caused by a similar virus, the Heartland virus, were reported in the United States.^5^ Another novel phlebovirus, the Hunter Island Group virus, was isolated from ticks in Australia.^6^

Although human-to-human transmission has been reported in China and South Korea,^7^–^9^ SFTS is transmitted mainly by ticks; Ixodidae ticks, in particular, are implicated as vectors of SFTSV.^1^,^10^,^11^ A previous study in South Korea reported that the minimum infection rate of SFTSV in *Haemaphysalis longicornis* ticks, major vectors of SFTSV, was 0.46%.^12^ In another study in South Korea, SFTSV was also detected in ticks that had bitten humans.^10^ From these studies, it is easy to see that SFTSV is common throughout the country. In this study, we aimed to evaluate the prevalence of SFTS in South Korea during 2013–2015, and to describe the clinical and epidemiologic features of this disease.

In China, approximately 3,500 cases of SFTS have been reported during 2014, with a case fatality rate ranging from 7.8% to 12.2%. In Japan, 170 cases of SFTS have been reported as of February 24, 2016; 46 cases (27%) were fatal at the time of notification (National Institute of Infectious Diseases, Tokyo, Japan).

Since the first reported case of SFTS in South Korea in 2013, between 2013 and 2015, from 207 hospitals throughout the country, we collected 1,697 serum samples from hospitalized patients who experienced symptoms of SFTS, such as high fever (≥ 38°C), vomiting, diarrhea, and/or fatigue, and demonstrated laboratory findings of SFTS, such as thrombocytopenia and/or leukopenia. Currently, in South Korea, enzyme-linked immunosorbent assay to detect IgM antibody is being developed, and the genetic diagnosis is used as the official diagnosis. All specimens were tested by using reverse transcriptase polymerase chain reaction (RT-PCR) using viral RNA that was extracted from the patients' sera by using the QIAamp Viral RNA Mini Kit (Qiagen, Inc., Valencia, CA) according to the manufacturer's instructions. Briefly, 140 μL serum was mixed with 560 μL QiaAmp Viral Lysis buffer (containing carrier RNA) and incubated for 10 minutes at room temperature. After adding 560 μL of 100% ethanol, the mixture was vortexed and applied to a spin column. After washing, RNA was eluted in 60 μL RNase-free water. To detect the SFTSV M segment gene, RT-PCR was performed by using the DiaStar 2X OneStep RT-PCR Premix Kit (Solgent Co., Ltd., Daejeon, South Korea) according to a previously described method.^10^

We identified 36 laboratory-confirmed cases of SFTS in 301 serum samples (12.0%) in 2013, 55 cases in 563 samples (9.8%) in 2014, and 79 cases in 833 samples (9.5%) in 2015. In South Korea, there are other diseases with similar symptoms as SFTS, such as hemorrhagic fever with renal syndrome, scrub typhus, and leptospirosis. We believe that the undiagnosed cases may be attributable to these other diseases. Although the positive rate decreased every year, these results demonstrate that the annual number of SFTS cases increased steadily. In addition, of the 170 cases, 54 deaths were confirmed to be associated with SFTS, showing an overall fatality rate of 32%. Annual case fatality rates exhibited a downward trend from 47% in 2013 to 27% in 2015 (Figure 1A

**Figure 1.**
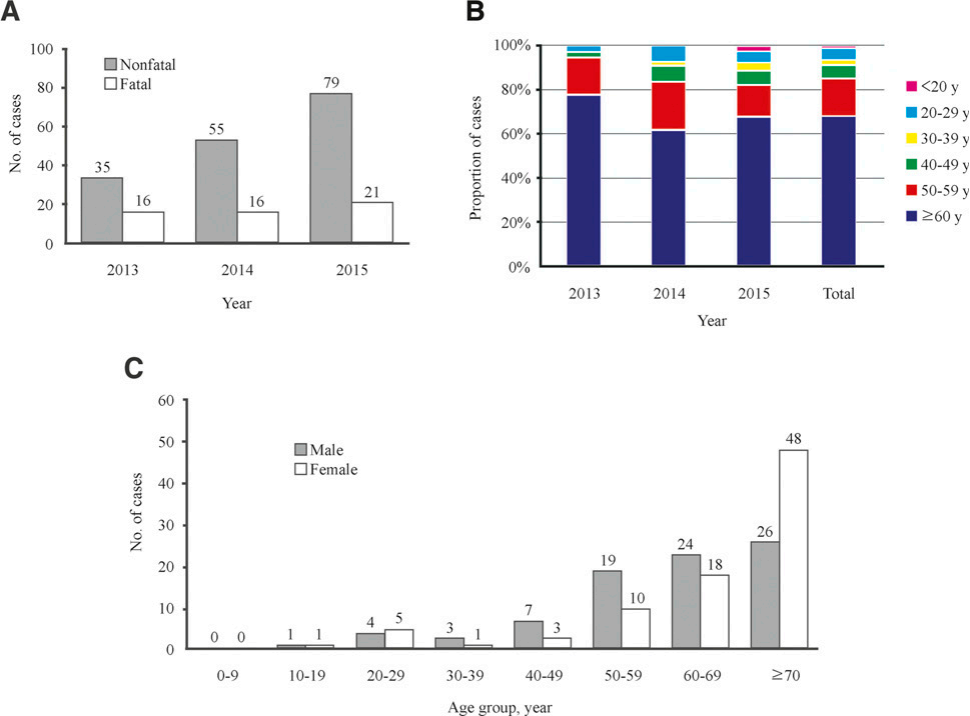
Distribution of severe fever with thrombocytopenia syndrome (SFTS) cases in South Korea during 2013–2015. (**A**) Number of SFTS cases per year. (**B**) Proportion of SFTS cases according to age. (**C**) Distribution of SFTS cases according to sex.

). We supposed that the fatality rate decreased every year due to more cases identified by surveillance as well as more patients successfully treated with plasma exchange and ribavirin.^13^

We found that the majority of SFTS cases were older patients. Of the 170 cases, 116 (68.2%) were aged ≥ 60 years. The median age of the patients was 64 years (range, 10–89 years), and the majority were aged ≥ 50 years (145, 85.3%) (Figure 1B). The overall female:male ratio was 1.02:1 (86/84). Interestingly, in age groups 50–59 years and 60–69 years, men had a higher rate of SFTS than women (11.2% versus 5.9% and 14.1% versus 10.6%, respectively; both *P* < 0.005). However, in the age group ≥ 70 years, women had a higher rate than men (28.2% versus 15.3%, *P* < 0.001) (Figure 1C).

We found that SFTS cases began to appear in April and ended in November. The highest number of cases was reported in July followed by October. The death toll increased from May until July and then gradually decreased (Figure 2A

**Figure 2.**
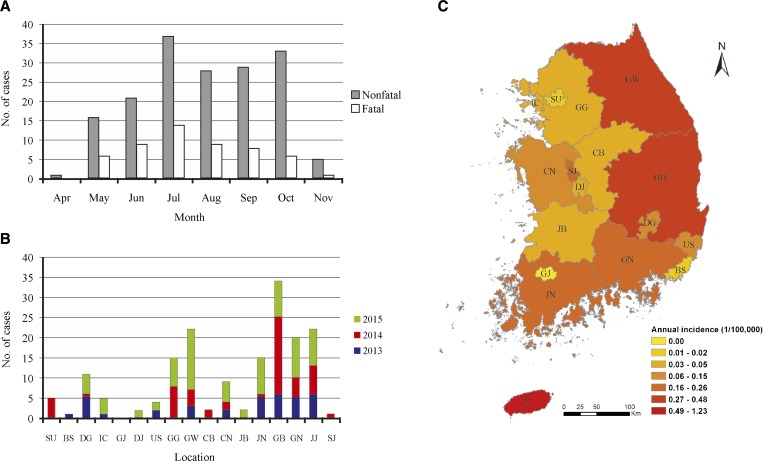
Temporal (**A**) and geographical (**B**, **C**) distributions of severe fever with thrombocytopenia syndrome (SFTS) cases in South Korea during 2013–2015. (**A**) Distribution of SFTS cases according to month. (**B**) Distribution of SFTS cases according to region. (**C**) Average annual incidence of SFTS. Map was created by using ArcGIS 10.3.1 (Esri, Redlands, CA). Abbreviations: BS = Busan City; CB = Chungcheongbuk Province; CN = Chungcheongnam Province; DG = Daegu City; DJ = Daejeon City; GB = Gyeongsangbuk Province; GG = Gyeonggi Province; GJ = Gwangju City; GN = Gyeongsangnam Province; GW = Gangwon Province; IC = Incheon City; JB = Jeollabuk Province; JJ = Jeju special autonomous Province; JN = Jeollanam Province; SJ = Sejong City; SU = Seoul City; US = Ulsan City.

).

We found that SFTS occurred mainly in the southeast part of the country. The highest number of cases was reported in Gyeongsangbuk Province (34/170, 20%) followed by Gangwon Province (22/170, 12.9%), Jeju special autonomous province (22/170, 12.9%), and Gyeongsangnam Province (20/170, 11.8%) (Figure 2B).

In this study, we provided an overview of the epidemiologic features and prevalence of SFTS in South Korea during 2013–2015. During this 3-year period, we detected SFTSV in 170 of 1,697 serum samples by using RT-PCR. Of the 170 cases, the primary symptoms included fever (80%), gastrointestinal symptoms (24.8%), thrombocytopenia (77%), and leukopenia (63.3%), which are similar to those reported in Chinese and Japanese patients.^2^,^14^ The clinical symptoms of 14 cases were not investigated.

To isolate SFTSV from the 170 serum samples, subconfluent monolayers of Vero E6 cells were inoculated with the sera. After three blind passages in new monolayers of Vero E6 cells, we examined the Vero E6 cells for presence of SFTSV by using RT-PCR. Consequently, via this method, we isolated 96 SFTSVs.^15^ Some of the isolates (isolated during 2013–2014) were sequenced and registered in GenBank (accession nos. KF282701, KF282702, KJ739543–KJ739592, and KU532917–KU532966). The other isolates (isolated in 2015) are still being prepared for genomic sequencing. The nucleotide sequences of these viruses were not only identified as SFTSV according to phylogenic analysis but also belonged to the Korean strains that were reported in a previous study^4^ (Figure 3

**Figure 3.**
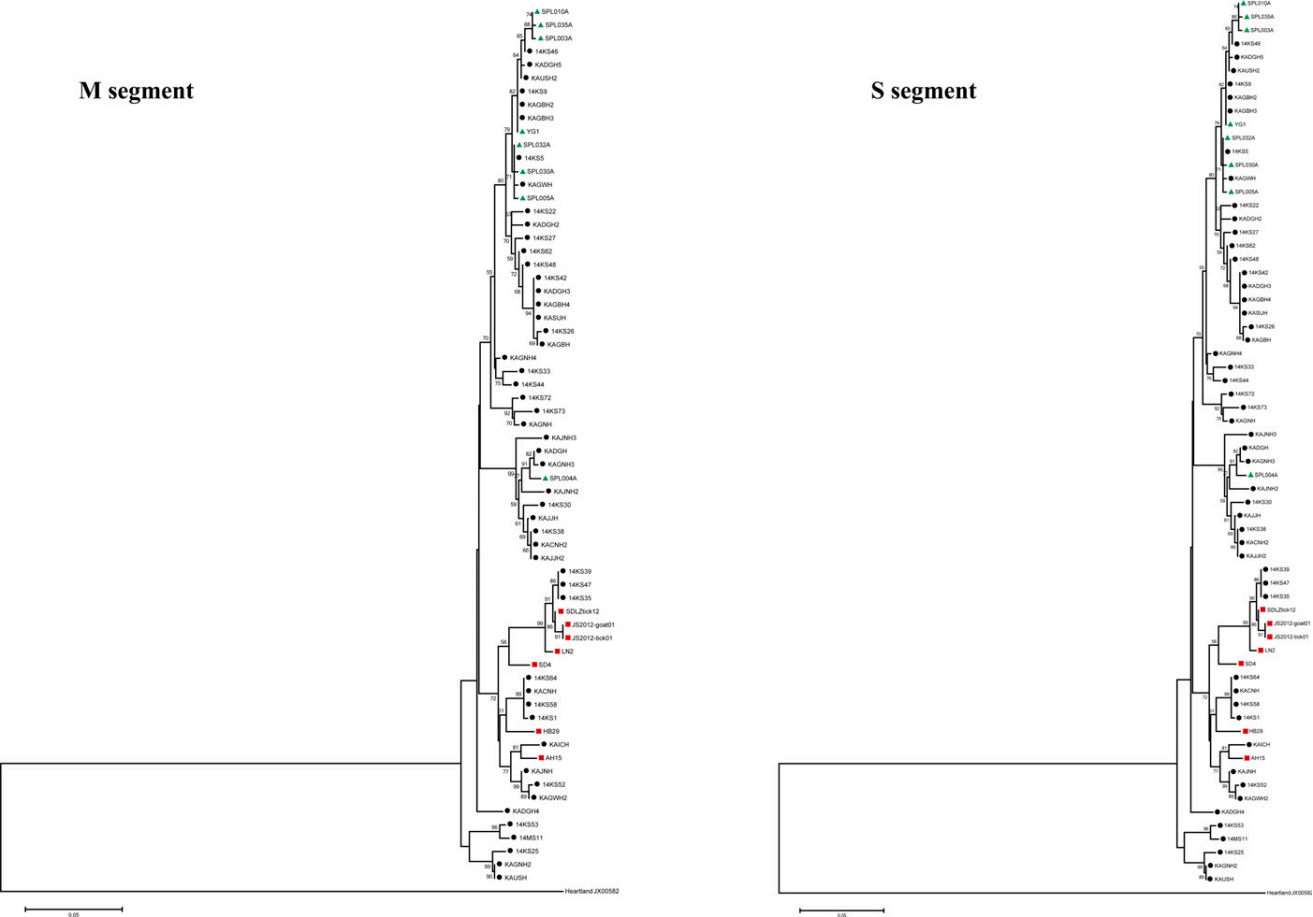
Phylogenetic analysis of severe fever with thrombocytopenia syndrome virus (SFTSV) Korean isolates based on partial M and S segment sequences. The phylogenetic trees were generated from aligned nucleotide sequences of 51 isolates of phleboviruses, including the identified SFTSV, by using MEGA version 5.2 software (Tempe, AZ). The Heartland virus was used as the outgroup. The sequences were analyzed by using the neighbor-joining method based on the maximum composite likelihood model. The minimal length trees were supported as the majority rule consensus tree in 5,000 replicates. The bootstrap replicates supporting each node are indicated. The Korean, Chinese, and Japanese isolates are indicated by black circles, red squares, and green triangles, respectively.

).

SFTS occurs mainly from May through August, the main activity time of the tick, which is generally considered to be the vector of SFTSV.^16^ Interestingly, incidence of SFTS slightly increased again in September and October in South Korea, unlike in China^17^ (Figure 2A). In South Korea, thanksgiving occurs during this period. At this time, people visit the graves of immediate ancestors to trim plants, clean the surrounding area, and offer food and drink to their ancestors. Therefore, we suggest that there may be exposure to these vectors through weeding a grave during the thanksgiving period.

Annual incidence of SFTS varied from 0 to 1.23 per 100,000 individuals, and had an average of 0.11 per 100,000 individuals nationally. The geographic distribution of annual SFTS incidence is displayed in Figure 2C. Jeju special autonomous province appeared to have the highest incidence with 1.23 per 100,000 individuals, followed by Gangwon, Gyeongsangbuk, Jeollanam, and Gyeongsangnam provinces. These provinces are located around the Taebaek and Sobaek Mountains in the eastern and southern parts of the country. Therefore, aside from Gangwon Province, the average temperature is higher than in other regions. Although ticks that carry the SFTSV are known to be distributed throughout the country,^12^ this fact does not correspond to local patient incidence. To investigate regional differences, future ongoing monitoring and epidemiologic studies are required.
